# Une polypose naso-sinusienne révélant une mucocèle du sinus maxillaire

**DOI:** 10.11604/pamj.2017.26.28.11373

**Published:** 2017-01-18

**Authors:** Moncef Sellami, Abdelmonem Ghorbel

**Affiliations:** 1 Service d’ORL et de Chirurgie Cervico-faciale du CHU Habib Bourguiba, Sfax, Tunisie

**Keywords:** Polypes nasaux, sinus maxillaire, mucocele, Nasosinusal polyposis, maxillary sinus, mucocele

## Image en médecine

La mucocèle est une formation pseudo kystique bénigne qui se développe rarement au niveau du sinus maxillaire. La mucocèle est secondaire à des situations diverses ayant en commun une inflammation et une obstruction de l’ostium du sinus. Cette dernière peut être primitive mais souvent secondaire à un (traumatisme, tumeur, inflammation chronique). Le diagnostic repose sur l’imagerie. La tomodensitométrie est indispensable pour apprécier les modifications osseuses et planifier la chirurgie endonasale sous contrôle endoscopique et l’IRM permet de préciser les rapports de la mucocèle. La prise en charge thérapeutique de ces mucocèles doit dans un même temps opératoire réaliser une marsupialisation de la mucocèle et traiter le facteur local en cause. Nous rapportons le cas d’un homme âgé de 21 ans, sans antécédents pathologiques notables qui consulte pour obstruction nasale bilatérale associée à une anosmie. L’examen clinique a trouvé une douleur exquise de la paroi antérieure du sinus maxillaire, une polypose nasosinusienne bilatérale et une voussure au niveau du palais osseux. La tomodensitométrie du massif facial a montré une masse hypodense homogène bien limitée ne prenant pas le produit de contraste comblant tout le sinus maxillaire gauche associée à une lyse osseuse régulière de ses parois et un comblement éthmoïdo-nasal bilatéral. Le patient a été opéré par voie endonasale et a eu une éthoïdectomie fonctionnelle associée à un abord du sinus maxillaire par méatotomie moyenne permettant la marsupialisation de la mucocèle. Les suites opératoires étaient simples sans récidive.

**Figure 1:**
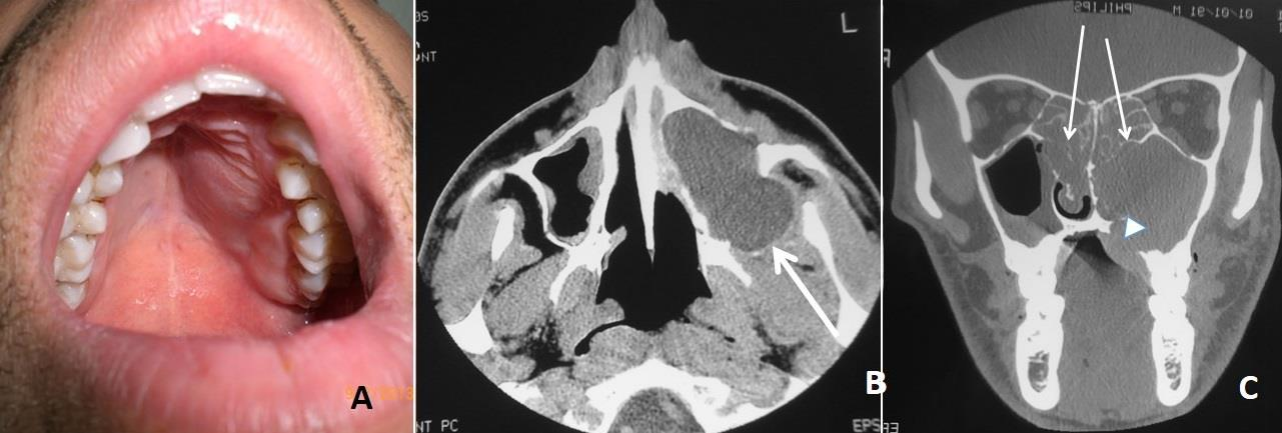
A) tuméfaction de l’hémi-palais osseux gauche; B) tomodensitométrie du massif facial en coupe axiale montre une masse hypodense du sinus maxillaire gauche avec lyse de la paroi postéro-latérale (flèche); C) tomodensitométrie du massif facial en coupe coronale montre un comblement éthmoido-nasal bilatéral (flèches) et la lésion sinus maxillaire qui érode le plancher du sinus (tête de flèche)

